# Adsorption, Adhesion, and Wettability of Commercially Available Cleansers at Dental Polymer (PMMA) Surfaces

**DOI:** 10.3390/ma17194755

**Published:** 2024-09-27

**Authors:** Stanisław Pogorzelski, Paulina Janowicz, Krzysztof Dorywalski, Katarzyna Boniewicz-Szmyt, Paweł Rochowski

**Affiliations:** 1 Institute of Experimental Physics, Faculty of Mathematics, Physics and Informatics, University of Gdańsk, Wita Stwosza 57, 80-308 Gdańsk, Poland; stanislaw.pogorzelski@ug.edu.pl (S.P.); p.janowicz.558@studms.ug.edu.pl (P.J.); krzysztof.dorywalski@ug.edu.pl (K.D.); 2 Department of Physics, Gdynia Maritime University, Morska 81-87, 81-225 Gdynia, Poland; k.boniewicz@wm.umg.edu.pl

**Keywords:** cleansers, dental materials, surface activity, Gibbs’ adsorption, adhesion, wettability-contact angle, surface energy, spreading kinetics

## Abstract

This study aims to evaluate the adsorptive, adhesive, and wetting energetic properties of five commercially available cleansers in contact with model dental polymer (PMMA). It was assumed that the selected parameters allow for determining the optimal concentration and place of key component accumulation for antibacterial activity in the bulk liquid phase and prevention of oral plaque formation at the prosthetic material surface. The adsorptive (Gibbs’ excesses *Γ_LV_*, critical micellar concentration) and thermal (entropy and enthalpy) surface characteristics originated from surface tension *γ_LV_(T)* and *γ_LV_(C)* dependences. The surface wetting properties were quantified upon the contact angle hysteresis formalism on the advancing *Θ_A_*, receding *Θ_R_* contact angles, and *γ_LV_* as the input data, which yield a set of wettability parameters: 2D adsorptive film pressure, surface free energy with its dispersive and polar components, work of adhesion, and adhesional tension, considered as interfacial interaction indicators. In particular, molecular partitioning *K_p_* and *Γ_LV_* are indicators of the efficiency of particular active substance accumulation in the volume phase, while *γ_SV_*, *a = Γ_SL_*/*Γ_LV_*, and *W_A_* point to the degree of its accumulation at the immersed polymer surface. Finally, the liquid penetration coefficient *PC* and the Marangoni temperature gradient-driven liquid flow speed were estimated.

## 1. Introduction

To attain satisfactory medical and visual effects, dentures should be immersed in the appropriate solutions for a certain period of time. These mixtures typically include a combination of several active compounds, like antiseptics, enzymes, surfactants, and diluted acids. For an effective denture cleanser, a chemical composition should guarantee a reduction of biofilm accumulation, being antibacterial/antifungal, non-toxicity, easy-to-use, and cost-effective [1]. Moreover, it should be capable of spreading over the surface of the denture to allow the contact area to be the highest and to make the contact angle (CA) lower, in order to be effective on smooth surfaces [2], and be able to penetrate well into interproximal areas and gingival pockets. The liquid penetration efficiency into denture capillaries and pores can be quantified by the penetration coefficient (*PC*) mediated by the surface tension of liquid, its viscosity apart from CAs on the substratum surface [3].

The chemical compositions of commercially available denture cleaners vary widely from each other, but the actual concentrations of compounds are rarely listed on the packaging. Therefore, only comparative analyses to demonstrate which formulations seem to be the most effective are possible and need to be updated as new modified products are promoted or formulations are proposed. In general, it is impossible to determine whether the inclusion of the antiseptic is of relevance to the findings of this study. It is essential to develop a common analytical scheme for the evaluation of the physico-chemical properties of denture cleaners upon their adhesive and spreading properties.

Denture appliances create room for microbial colonization and multispecies microbial biofilm formation. The biofilm (or dental plaque) is understood as a compact microbial community layer fixed in a polymeric matrix [4]. Denture biofilms appear to be a various pathogens source, for instance, *Streptococcus mutans*, *Candida* spp., *Escherichia coli*, and *Staphylococcus aureus* [5]. It is expected that microbes residing in biofilms seem to be more resistant to antimicrobial substances than being in a planktonic form. As an example, it was demonstrated in [6] that a shorter exposure period was needed to eliminate all tested planktonic phase bacteria and *Candida* in reference to the residing ones in the biofilm form. Moreover, these findings revealed that the immersion time increase is an important factor that allowed the substances to efficiently diffuse into and concentrate in the biofilm structure (so-called target location), leading to an intensified anti-biofilm action.

The aim of this study was to predict the efficacy of five denture cleansers on multi-species microbial biofilm creation based solely on physical attributes, i.e., by analyses of adhesion, adsorption, and wettability energetics in a polymer/cleanser interfacial system [7]. The model polymer substratum studied here, polymethylmethacrylate (PMMA), known for its excellent biocompatibility and resistance to aging processes, is commonly used in acrylate plastics. Since PMMA stands for a weakly polar polymer material that contains -CO, -OCH_3_, and -CH_3_ polar groups, several mechanisms characterize the adsorption at PMMA surfaces interacting with the surfactant-containing liquids [8].

Our pilot studies could be useful in the targeted treatment of removable dental dentures with cleaning fluids understood as delivering its antibacterial and antifungal components at the highest possible concentration to the place where its action will be most effective, i.e., in a (bulk) planktonic of the solution phase or in the surface phase adhered on the solid polymer, respectively. The successful concept realization requires the functional correlation between the adhesion–adsorption–wettability parameters and the viability of the oral plaque microbes to be established, as studied in [6].

**Figure 1:**
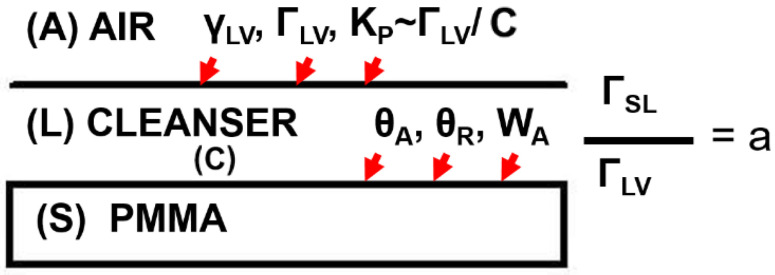
Selected adsorptive, adhesive, and wettability parameters corresponding to the particular interfacial region characterization for model dental polymer PMMA–cleanser system. Denotations: liquid/vapor (*LV*) and solid/liquid (*SL*) interfaces; *γ_LV_*—the surface tension; *Γ_SL_*/*Γ_LV_ = a*—the Gibbs excesses ratio (=d(*γ_LV_* cos *θ_A_*)/d*γ_LV_*)); *C*—the bulk concentration; *K_P_* = *RT (Γ_LV_*/*C)*—the partitioning coefficient of molecules between surface and bulk phases; *θ_A_*, *θ_R_*—the dynamic contact angles (CA): advancing and receding, respectively; the contact angle hysteresis *CAH* = *θ_A_* − *θ_R_*; *W_A_*—the adhesion work; *T*—the absolute temperature, and *R*—the gas constant.

The selection of the adsorption, adhesion, and wettability parameters adapted to characterize interfacial regions in the model vapor/cleanser/PMMA system is schematically shown in Figure 1.

The wettability parameters of model surfaces analyzed here, solid surface free energy (SFE) *γ_SV_*, its dispersive $\gamma_{SV}^{d}$ and polar $\gamma_{SV}^{p}$ components, adhesive film pressure *Π*, work of adhesion (*W_A_*), spreading (*W_S_*), and cohesion (*W_C_*), and their evolution due to the surface treatment procedures, were evaluated by means of (*CAH*) formalism presented in [9]. This approach allows the parameters of surface wettability energetics to be determined from the following quantities: the surface tension of the probe liquid *γ_LV_* and the dynamic contact angles, *Ѳ_A_*, *Ѳ_R_*, advancing and receding, respectively, that is, the only formalism taking into account the role played by the adsorbed layer at the solid surface. All the remaining interfacial parameters discussed here are derived from *γ_LV_(C)* and *γ_LV_(T)* dependences and are defined and discussed in detail in our previous work [7] and briefly summarized below. Recently, the wettability studies performed on metal surfaces covered with sprayed paint layers revealed the decisive role played by the roughness architecture of the substratum, with the strength of liquid/solid interactions [10].

## 2. Theoretical Principles

### 2.1. Adsorptive and Thermodynamic Surface Characteristics

The adsorption of surfactants at the liquid/vapor interface, referred to as the limiting area of the adsorbed molecules *A_lim_ = kT*/*Γ_max_*, is given by the Gibbs’ equation [11]:

(1) $$\Gamma =-\frac{1}{RT}\frac{d{(\gamma }_{LV})}{d(lnC)}.$$

The surface activity *σ* of a surfactant in the solution, proportional to the partitioning coefficient *Kp = Γ*/*C* of the surfactant molecules between the surface and bulk phases (*C < CMC*-critical micellar concentration), is defined as

(2) $$\sigma =\Gamma \frac{RT}{C}=-\frac{d(\gamma_{LV})}{dc}.$$

Gibbs’ excesses ratio a (see Figure 1) is defined as

(3) $$\frac{d(\gamma_{LV}cos\theta )}{d(\gamma_{LV})}=\frac{\Gamma_{SV}-\Gamma_{SL}}{\Gamma_{LV}}.$$

Surface entropy (*S_S_*) and total surface enthalpy *H_S_* (=*γ* − *TS_S_*) can be expressed as

(4a) $$S_{S}=-\frac{d{(\gamma }_{LV})}{dT},$$

(4b) $$H_{S}=\gamma_{LV}-T\frac{d{(\gamma }_{LV})}{dT}.$$

### 2.2. Surface Wettability Energetics

The solid surface free energy (*SFE ≡ γ_SV_*) and the related parameters can be obtained from the following dependencies.

Adsorbed matter 2D film pressure, *Π* = *γ_LV_*(cos *θ_R_* − cos *θ_A_*) and *γ_SV_*, are related to each other via

(5) $$\gamma_{SV}=\frac{\Pi {\left(1+cos\theta_{A}\right)}^{2}}{{\left(1+cos\theta_{R}\right)}^{2}-{\left(1+cos\theta_{A}\right)}^{2}},$$

while the other quantities, the work of adhesion, $W_{A}=\gamma_{LV}(1+cos\theta_{A})$, and the work of cohesion, $W_{C}=2\gamma_{LV}$, yield the work of spreading $W_{S}=W_{A}-W_{C}$, which demonstrate a relation between the wettability and the adhesion mechanical strength. As such, it allows the competition between solid–liquid adhesions for different liquids to be quantified [12].

### 2.3. Kinetics of Spreading and Penetration

The penetration coefficient (*PC*) quantifies the liquid penetration process into capillaries and pores of the substratum [3]. It can be determined from *γ_LV_*, the viscosity *μ* of the liquid, and the liquid/polymer CA:

(6) $$PC=\frac{\gamma_{LV}}{2\mu }cos\theta ,$$

where μ denotes the dynamic viscosity.

The speed $U_{S}$ of the Marangoni effect attributed to the temperature gradient-induced liquid flow in a layer of *d* thickness reads as follows [13]:

(7) $$U_{S}=\frac{d}{4\mu }S_{S}\nabla T.$$

Our previous work on the adsorptive and adhesive signatures of the mouthrinse mixture/PMMA prosthesis polymer system revealed that the molecules of the antibacterial component (e.g., paraben, for instance) should be present in a high concentration in the fluid bulk phase in the oral cavity (high availability of the bactericidal agent in the bulk phase) [7]. This means that the partitioning coefficient *K_P_* should be as low as possible. On the other hand, measurements using confocal microscopy showed that effective elimination of the dental plaque formation at the submerged polymer material of a dental prosthesis requires obtaining high adhesion of the active substance to the polymer surface (i.e., at the L/S interface surface), and this requires obtaining a high strength of interaction between a liquid in contact with a polymer, i.e., with high adhesion work, low surface energy, low 2D pressure, and low surface excesses ratio (*a*) values [6].

It is intended that the same research methodology, performed on cleanser/PMMA systems in the framework of this work, will confirm that a variability of the adhesive and wetting parameters of dental denture cleaning fluids would be a novel indicator of the deposited biological and mineral material removal effectiveness and allow for the optimization of their chemical composition.

## 3. Materials and Methods

A model dental polymer substratum PMMA was used in the form of flat plates (20 × 20 mm) (Organika S.A. Sarzyna, Poland), further traded in a 20% methanol solution, placed in an ultrasonic cleaner, and finally rinsed with Milli-Q water. In order to reduce the residual monomer content, polymer samples were kept in distilled water for 40 h. The material characterization study concerned the evaluation of basic physical properties of five commercially available cleansers: density ρ, dynamic viscosity μ, surface tension *γ_LV_*, acidity (pH), and contact angles (CA) when in contact with solid surface. Detailed descriptions of the apparatus characteristics and measuring procedures are presented in detail elsewhere [7]. Briefly, they are given below. A tensiometer (model: PI-MT1M, Donserv, Warsaw, Poland) based on de Nuoy ring method (a 6 cm circumference ring made of platinum–iridium, Sinterface, Berlin, Germany), with an accuracy of 0.1 mN m^−1^, was used to measure the surface tension of the studied liquids. The Ubbelohde viscometer (Equimed, Krakow, Poland) was used for the kinematic viscosity measurements. The pH measurements of the model liquids were carried out with a pH meter (CP-315, Elmetron, Zabrze, Poland) with a universal electrode. Distilled water (as a reference) was taken from a water deionization apparatus (Millipore, conductivity 0.05 μS cm^−1^) with pH = 6.8, and surface tension *γ_LV_* = 72.5 mN m^−1^. The measurements were performed at room temperature *T* = 25 °C unless stated otherwise. The solutions of a cleanser were obtained by dissolving one tablet of each product in 200 mL of distilled water (45 °C) following the manufacturer’s instructions. The polymer samples were flushed in distilled water after a 5 min lasting cycle. All the experimental parameters were determined upon averaging over sets of 6–10 single measurements for each cleanser–polymer system.

According to the producer data sheets, the compositions of the cleansers studied are as follows:

- Corega Tabs: sodium bicarbonate, citric acid, potassium caroate, sodium carbonate, sodium carbonate peroxide, TAED, sodium benzoate, PEG-180, sodium lauryl sulfoacetate, PVP/VA copolymer, aroma, subtilisin, CI 42090, CI 73015;
- Fitty Dent: sodium bicarbonate, sodium carbonate peroxyhydrate, trisodium phosphate, potassium monopersulphate, sulfamic acid, sodium perborate monohydrate, PVP, sodium lauryl sulfate, TAED, aroma, CI 42090;
- Kin Oro: sodium bicarbonate, potassium caroate, citric acid, sodium carbonate, sorbitol, PVP/VA copolymer, sodium lauryl sulfate, sodium lauryl sulphoacetate, aroma, CI 73015;
- Kurikur: sodium bicarbonate, potassium caroate, sodium carbonate, TAED, sodium carbonate peroxide, sodium sulfate, malic acid, PEG-90, PEG-150, citric acid, sodium dodecylbenzenesulfonate, aroma, CI 28440, CI 42090;
- Protefix: sodium persulfate monohydrate, sodium bicarbonate, citric acid, sodium carbonate, sorbitol, PVP/VA copolymer, sodium lauryl sulfate, sodium lauryl sulfoacetate, aroma, CI 73015.

The physical and surface characteristics of the studied cleanser solutions are collected in Table 1.

All the studied solutions are capable of significantly lowering water surface tension to 33.4 (Fitty Dent)—36.2 (Corega Tabs) mN m^−1^, thus exhibiting their highly surface-active properties. pH values are contained in the range of 7.8 (Kin Oro) to 9.1 (Protefix); i.e., the solutions are alkaline.

PMMA substrata were placed in a humidity-controlled cell (see Figure 1 in [14]) to perform CA measurements. The inclined plate method was chosen to determine dynamic CA by analyses of the side-view images of sessile drops placed at the solid substrata with ImageJ program. The sessile drop shape profile analysis was adapted to evaluate CAs from the sessile drop (ranging in 4–6 mm in diameter) images. Exemplary images of such registrations are shown in Figure 1 of [7]. The relation between Young’s equilibrium CA and the dynamic contact angles was applied here: cos *θ* = ½ cos *θ_A_* + ½ cos *θ_R_*, after [10]. Details on the CA measuring procedure and data evaluation can be found in [7]. The surface tension vs. temperature dependence (within the range of 25–45 °C) was obtained using the glass vessel containing the cleanser solution placed in a temperature-controlled box. The surface tension *γ_LV_* vs. cleanser concentration dependences were obtained from surface tension measurements on the subsequently diluted original samples and plotted as a function of the normalized (with respect to the starting *C*_0_) concentration *C_n_ = C*_0_/*n*, where: n = 2, 3…

## 4. Results and Discussion

### 4.1. Surface Adsorption at Vapor/Liquid Interface

**Figure 2:**
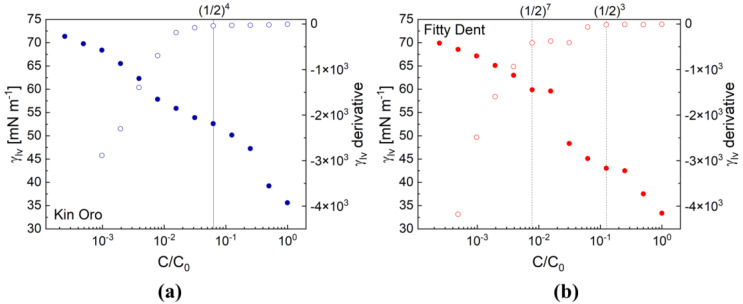
Surface tension and surface tension derivative of Kin Oro (**a**) and Fitty Dent (**b**) solutions vs. relative concentration *C*/*C*_0_ plot. The lines point to the critical value *C*/*C*_0_ = 0.062 (Kin Oro) and 0.064 (Fitty Dent) corresponding to *CMC*. The best-fit straight-line approximation procedures to the data were used to determine the inflection point. In this system (**b**), a smooth discontinuity of the *γ_LV_*(*C*) plot can be first noticed (*C* < *CMC*; around 0.125) at the so-called critical aggregation concentration (*CAC*), then followed by another one at *CMC*, where regular micellar structures are expected to appear [11]. Denotations: experimental data for *γ_LV_*(*C*/*C*_0_) ● Kin Oro, ● Fitty Dent; the surface tension derivative = σ, d*(γ_LV_)*/d(*C*/*C*_0_), ◦ Kin Oro, ◦ Fitty Dent.

The principal surface adsorption parameters for cleanser formulations derived from a surface tension *γ_LV_* and its derivative, i.e., d*(γ_LV_)*/d(*C*/*C*_0_) vs. normalized concentration *C*/*C*_0_ plot, as shown in Figure 2a (Kin Oro) and Figure 2b (Fitty Dent), are all collected in Table 2. Such a concentration scaling approach for samples of largely unknown composition yields a universal measure of a water mixture σ referred to as the original (initial) sample concentration *C*_0_.

The lowest *CMC* value was noticed for Kin Oro. For the remaining cleansers, the *CMC* ranged between 0.043 and 0.088, which is significantly lower than for the saliva-forming substance mixture (0.31, [7]). The data obtained revealed that the recommended by producer concentrations to apply in a patient practice, *C*_0_, are higher by at least an order of magnitude than the ones required to obtain the saturated adsorption layer condition (*C = CMC*). For higher concentrations (*C >> CMC*), the micellar solubilization mechanism is activated; i.e., the micellar aggregates formed in the bulk are capable of entrapping insoluble substances [11]. The probes analyzed here lowered the water surface tension (=72.5 mN m^−1^) by approximately 20–30 mN m^−1^. For example, the surface effectiveness *γ_CMC_* of the model substances widely used in the physical chemistry of surfaces are [11] ~44 (anionic SDS (sodium dodecyl sulfate)), ~35 (cationic DTAB), and ~32 mNm^−1^ (non-ionic C_12_E_m_). Other adsorptive parameters, surface activity *σ* (slope of the *γ_LV_(C)* below *CMC*) and the corresponding *A_lim_* related to *Γ_max_*, were evaluated as well. The *Γ_max_* values were contained in the range from 1.16 (for Corega Tabs) to 4.71 (Protefix)·10^6^ mol cm^−2^, which corresponded to the *A_lim_* varying from 35.2 to 140.1 nm^2^ molec.^−1^; for instance, the *A_lim_* values registered for medical liquids (glycerol, glycols) turned out to be lower by a factor 10^2^ (0.58–1.57 nm^2^ molec.^−1^), but saliva-formed films revealed an *A_lim_* (=12.6 nm^2^ molec.^−1^) lower up to the order of magnitude than the cleansers did on average [7]. From a medical point of view, it is important to achieve high enough concentrations of free preservative compounds (contained in cleanser formulations) in water to attain sufficient antimicrobial protection. It is understood that preservatives could remain more or less available for contact with microorganisms, depending on the place of their location, i.e., air/solution, solution/solid interfaces, or bulk water phase. The surface activity of the surfactant mixture σ is proportional to the partitioning coefficient *K_P_ = Γ*/*C*. The highest absolute values of the surface activity indicate that the said substance molecules are most hydrophobic and slightly soluble in the solution bulk. Kin Oro is the most hydrophobic and slightly soluble in bulk (the lowest *CMC* and the highest |*σ*|). On the other hand, Protefix seems to be of the highest solubility and a lower surface activity (a low *CMC*, relatively high *γ_CMC_*, and the lowest |*σ*|). Our surface tensometry studies performed on formulations of medical surfactants with drug preservatives exhibited that methylparaben had a *|σ|* value about 10 times lower than benzalkonium chloride (BAC) and turned out to be better soluble in the aqueous phase in the form of free molecules more available for the contact with microorganisms [15].

### 4.2. Surface Thermodynamics Parameters

**Figure 3:**
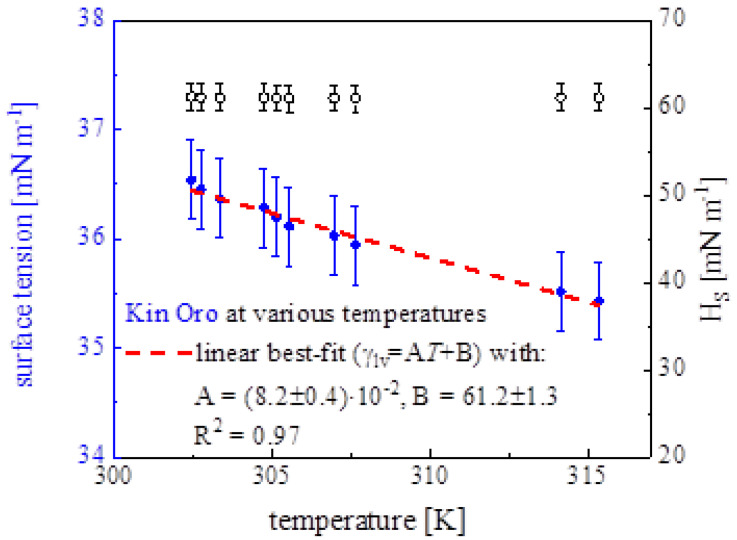
Surface free energy *γ_LV_* (blue closed symbols, ●) and enthalpy *H_S_* (black open symbols, *◦*) as a function of *T*, for Kin Oro solution (at *C = C*_0_). *S_S_* = (0.082 ± 0.04) mN m^−1^ K^−1^ obtained from the slope of the *γ_LV_(T)* relation linear fit to the data.

An exemplary surface tension *γ_LV_* versus temperature *T* dependence together with surface enthalpy *H_S_* variability, derived for Kin Oro, is presented in Figure 3. All the surface thermodynamics functions, for the studied cleansers, are collected in Table 3. A linear plot of *γ_LV_* (*T*) can be expressed with a dependence *γ_LV_* = −0.082*T* + 61.2. *H_S_* exhibited a constant value along a whole T range. Values of *S_S_* ranged from 0.061 (Kurikur) to 0.183 (Protefix) mN m^−1^ K^−1^, close to pure water (0.163) and saliva (0.11) data [7]. As a result, the most organized interfacial structure (lowest *S_S_* values) is formed by Kurikur, whereas Protefix creates layers of the lowest degree of organization (highest *S_S_*). The presence of the double electric layer effect in our system could lead to an interfacial system of particular thermodynamics [16]. The increase in surface adsorption of the surfactants leads to surface entropy decrease [17]. Generally, *H_S_* took lower values, for the cleansers, from 52.0 (Kurikur) to 87.8 (Protefix) mN m^−1^ if referred to the pure water (120.6) and saliva (85.6) as well [7]. *H_S_* as a temperature-independent quantity is more suitable to characterize interfacial system tension than γ_SV_. The entropic term *TS_S_* contributions to the surface enthalpy *H_S_* varied from 0.35 (Kurikur) to 0.62 (Protefix) and were higher if referred to the water (0.39) reference case, pointing to the presence of adsorbed species. In the studied water solution mixtures, a wide variety of components of differentiated chemical structures are present apart from proteins, carbohydrates, and glycerides of small surface activity, highly surface active free fatty acids, esters, and alcohols can be met. Their trace amounts can strongly affect the resultant complex surface adsorptive layer governed by the competitive adsorption mechanism. In such highly-structured, polymer-like aggregated systems, the thermodynamic and kinetic processes turn out to be particularly complex [18]. It was found that for Corega Tabs, Fitty Dent, Kin Oro, Kurikur, the adsorption process turned out to be entropy-controlled (*TS_S_* > *γ_LV_*), but the enthalpy-controlled (*TS_S_* < *γ_LV_*) process was revealed for Protefix, pure water, its derivatives (orange juice, Coca Cola), and saliva [7].

### 4.3. Contact Angles Analysis to Wettability Energetics

The static equilibrium contact angle *θ* values for the reference system PMMA–pure water (=64.6°) are significantly higher than for the cleanser liquids ranging from 28.2° (Protefix) to 40.0° (Corega Tabs)—see Table 4. We are concerned with hydrophilic surfaces (θ < 90°). The observed decrease in *θ* and *γ_SV_* as PMMA polymer is exposed to cleansers may be a result of a few processes: surface adhesion and absorption in solid pores, erosion, etching, and micro-roughness creation, leaching of the more soluble PMMA components, etc. Generally, the values of *γ_SV_* obtained are characteristic for surfaces of low SFE, such as for the PMMA/cleanser system ranging from 26.2 (Corega Tabs) to 31.9 (Kin Oro) mJ m^−2^, whereas contact with pure water led to *γ_SV_* = 38.9 mJ m^−2^. Such surface energies (20–30 mJ m^−2^) are found for hydrophobic substrata like polymers [11].

The *CAH* approach provides a set of parameters (collected in Table 4), allowing for the quantification of the surface interactions between a surfactant-containing liquid and polymeric substratum. In fact, contact angle hysteresis parameter deviations from a reference case (i.e., for an unaffected surface) rather than their absolute values stand for an effective tool for the surface modification tracing resulting from exposure to cleanser solutions. For the pure water/PMMA system, *CAH* = (24.0 ± 2.0)°, very similar to the data reported in the literature (=23.5° [19]). There are a few mechanisms responsible for the hysteresis: chemical heterogeneity, spatial hydrophobic heterogeneity, surface micro-roughness, drop size effect, adsorbed surfactant molecules reorientation, liquid penetration into solid pores, multilayer structures of interfacial layer, liquid evaporation, surface chemical reactions, etc. *CAH* values for the cleanser-treated surfaces range from 5.4 (Protefix) to 36.2° (Corega Tabs), which results from a surface activity diversity of the surface-active compounds forming the mixture since *θ_A_* (from 30.8° (Protefix) to 53.3° (Corega Tabs)) corresponds to the most hydrophobic component and *θ_R_* (from 12.5° (Kin Oro) to 25.4° (Protefix)) points to the less hydrophobic one [20]. The 2D film pressure *Π* (1.5 (Protefix)—12.2 (Corega Tabs) mN m^−1^) was several times lower than recorded for the PMMA surface contacted with saliva [7] and pure water (26.9 mN m^−1^), which seems to be an effect of competitive surface adsorption at the PMMA/liquid interface. Lower values for *Π* and *W_A_* indicate that the cleanser solutions spread over the surface and, resulting from the competitive adsorption mechanism, can modify the surface even further via the implementation of fewer surface-active species. From a thermodynamic point of view, only a surface modification process is possible, which is accompanied by a decrease in the system energy. As such, the surface adsorption of cleanser solutions at the PMMA substratum is considered the energetically favored process. Such a component can remove already adsorbed contaminants residing in the dental material. Since *W_A_ = γ_LV_ + γ_LV_* cos *θ_A_*, the second term (called the adhesional tension) contribution to the *W_A_* (i.e., ratio: *γ_LV_* cos *θ_A_*/*W_A_*) spanned from 0.38 (Corega Tabs) to 0.46 (Protefix) and attained only 0.11 and 0.02 for pure water and saliva, respectively [7]. The adhesional tension stands as an indicator of the adhesive strength removal ability of the cleanser liquids. It is equal to the difference of SFE between clean and liquid-coated substrata: γ_SL_ = γ_SV_ − γ_LV_ cos θ_A_.

**Figure 4:**
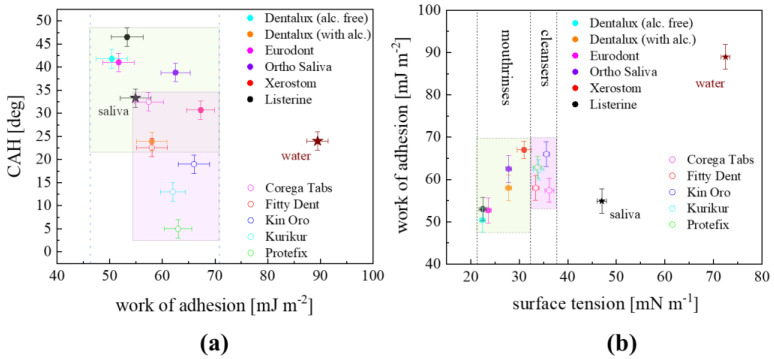
(**a**) Contact angle hysteresis *CAH* versus *W_A_*; (**b**) *W_A_* plotted versus *γ_LV_*, for PMMA-probe liquid systems at *C = C*_0_. Data for cleanser (open symbols) and mouthrinse (closed symbols) liquids, and water as a reference.

The diversity of the probe liquid’s polarity in contact with the PMMA surface can be demonstrated by the 2D distribution of the experimental data: *CAH* plotted versus *W_A_* and of *W_A_* versus *γ_LV_*, as shown in Figure 4a and Figure 4b, respectively.

As shown in Figure 4a, a rectangular (light blue) shaded region occupied the area of the coordinates of *CAH* (23.0–42.5°) and *W_A_* (42.1–67.8 mJ m^−2^) corresponding to the data for mouthrinse/PMMA surfaces. The cleanser/PMMA data points were placed in the different regions of the picture, of a rectangular shape (light rose-colored) with the following coordinates: *CAH* (5.4–32.6°) and *W_A_* (57.4–66.0 mJ m^−2^). However, the areas corresponding to each kind of polymer treatment liquid were overlapping to each other to some extent. Other water-derived mixtures (water, juices, cola) and pure, one-component surfactant solutions (despite their polarity and concentration) reside outside the specified regions as already revealed in [7], which demonstrated evidence of the particular adhesive signatures of both mouthrinse and cleanser formulations. Similarly, in Figure 4b, the cleanser-corresponding points were contained in a rather narrow region limited to *W_A_* ranging from 57.4–66.0 mJ m^−2^ with *γ_LV_* varying between 33.4 and 36.2 mN m^−1^, whereas the mouthrinse-corresponding ones were shifted to the region of generally lower *γ_LV_* between 29.7 and 38.7 mN m^−1^, with similar *W_A_* varied within 50.4–67.3 mJ m^−2^. The data boxes are separated from each other. It can be noticed that the remaining background (water, saliva) data points were located far outside the specific region [7]. Finally, the particular values of *CAH*, *W_A_*, and *γ_LV_* exhibited in CA studies could be useful in the selection of the most effective process liquid ingredients in dental material wettability adjustment recommendations.

**Figure 5:**
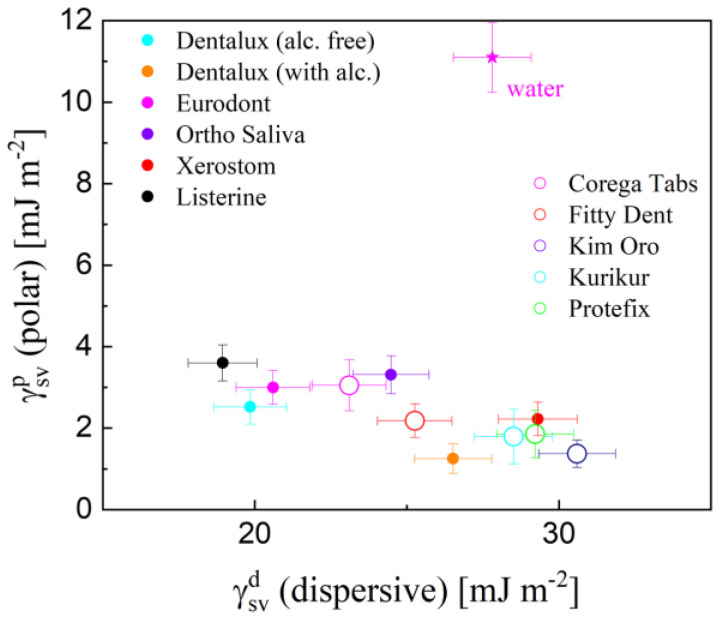
Polar $\gamma_{SV}^{p}$ versus dispersive $\gamma_{SV}^{d}$ terms of the total surface free energy *γ_SV_*, for the studied solutions in contact with PMMA substratum at *C = C*_0_. Additional data from mouthrinse studies are included in [7].

From the wettability-based surface energetics point of view, for the PMMA/water solution, dispersive interaction forces dominate since the dissipative term $\gamma_{SV}^{d}$ in the total solid surface energy *γ_SV_* accounts for around up to 89–98%. In particular, the dispersive term contributions to the total *γ_SV_* were varied between 0.88 (Corega Tabs)—0.96 (Kin Oro). Both contributions (polar and nonpolar/dispersive components) to the total surface free energy for the PMMA surface mediated by the cleaning liquids are shown in Figure 5.

Rather low $\gamma_{SV}^{p}$ values were obtained for cleansers (1.3 (Kin Oro)—3.1 (Corega Tabs) mJ m^−2^) comparable to those revealed by mouthrinses (1.3–3.6 mJ m^−2^) but apparently lower than found for the other liquids, such as water, juices, and saliva (4.54–11.1 mJ m^−2^) [7]. Values of the dispersive term $\gamma_{SV}^{d}$ were contained within a narrow range of 23.1 (Corega Tabs) and 30.6 mJ m^−2^ (Kin Oro), slightly higher than those represented by mouthrinses (18.92–29.31 mJ m^−2^). However, the general evolution trend of the $\gamma_{SV}^{p}$ versus the $\gamma_{SV}^{d}$ relation can be approximated with the following linear best-fit function: $\gamma_{SV}^{p}\;$= −0.18 (±0.02) $\gamma_{SV}^{d}$ + 6.81 (±0.31); R = 0.82. Several kinds of forces (originating from electrostatic, covalent, and hydrogen bonding interactions) mediate the interfacial behavior of surfactants. Steric forces result from molecules with long chain segments existing in the system, mostly polymers and surfactants [19]. Comprehensive results point to the single source of CAH origin: the difference in solid–liquid interaction in the vicinity of the contact line during advancing and receding movement modes [21]. CAH large values are indicative of stickiness. The increased interactions at the solid adsorbed film-solution drug the receding contact line leading to smaller *θ_R_* and larger *CAH*. If the *CAH* phenomenon is governed mainly by the interfacial liquid/solid interaction, it will manifest as adhesion. Moreover, adhesion is correlated to *θ_R_* only [22]. On the surface of PMMA, the -CH_3_, -CO, and -OCH_3_ functional groups are present, so surfactants can interact via a variety of mechanisms. In particular, the interaction between a surfactant molecule hydrophilic group and the polymer surface takes place via intermediary water molecules [23].

Finally, the variability of the wettability energetics parameters for Corega Tabs and Protefix showed that the values of these parameters took the borderline (smallest and largest) values throughout the entire range of their variability for a family of cleansers. These fluids seem to have dramatically differing adhesive properties, probably resulting from significantly different chemical compositions.

### 4.4. Cleanser Dissolution vs. Wettability

**Figure 6:**
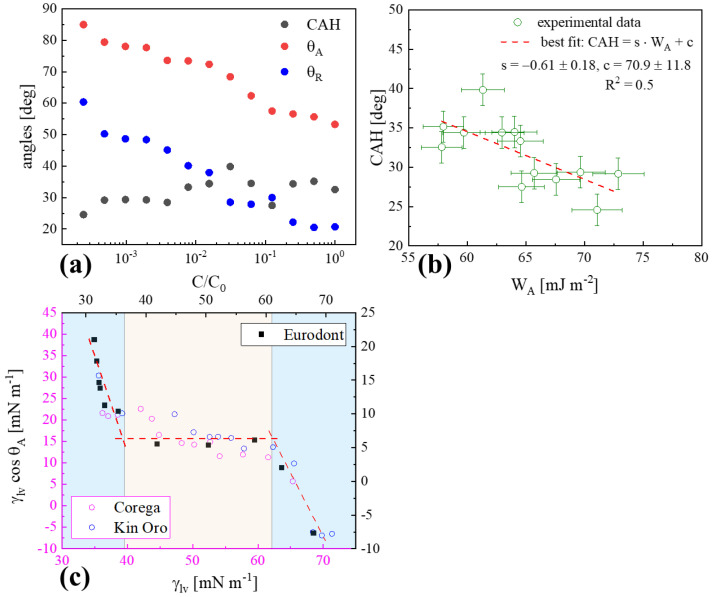
(**a**) Dynamic CAs and *CAH* versus relative concentration *C*/*C*_0_; (**b**) *CAH* as a function of *W_A_* for subsequent Corega Tabs dilutions; (**c**) adhesional tension *γ_LV_* cos *θ_A_* vs. surface tension *γ_LV_* leading to the surfactant excesses ratio, *Γ_SL_*/*Γ_LV_* = *a* (slope of the red dotted line) determination, for Corega Tabs and Kin Oro (open symbols) and Eurodont (black squares and right-hand scale); post-micellar (*C > CMC*) and pre-micellar (*C < CMC*) regions are marked in blue.

The cleanser product, Corega Tabs of initial concentration *C*_0_, was subsequently dissolved with ultrapure water and then used in CA studies of PMMA surface wettability. The values of *θ_A_*, *θ_R_*, and *CAH* as a function of the relative concentration are depicted in Figure 6a, while the whole list of the wettability parameters considered for such a system is summarized in Table 5.

As expected for a regular surface-active mixture [18], in the post-micellar region (*C > CMC*), no significant CAs and its *CAH*~const. (around 32.3°) variability was observed. For lower concentrations, i.e., the pre-micellar region, starting from *CMC*, a continuous increase in dynamic CA was noticed, *CAH* fluctuated around the lower constant value (~23.5–29.6°), the film pressure (*Π*) took two times higher (~27.7–32.0 mN m^−1^) values, and *W_A_* increased significantly to 61.3–72.9 mJ m^−2^ in reference to the post-micellar concentration range. The specific behavior originates from the viscoelastic reaction of the already adsorbed molecular film structure to the adhesional tension (=*γ_LV_* cos *θ_A_*), resulting from the cleanser application. A somewhat scattered *CAH* versus *W_A_* dependence, shown in Figure 6b, is approximated here with a linear dependence (R^2^ = 0.5) as CAH↓ and W_A_↑ with the serial dilution, and a liquid polarity ($\gamma_{SV}^{p}$) increase was accompanied by the almost constant *γ_SV_*. Since *W_A_* is a sum of *γ_LV_* and the adhesional tension *γ_LV_* cos *θ_A_* (decreased by a factor ~1.6), the observed W_A_↑ (*γ_LV_* ~ const. for *C < CMC*) could be an effect of the different adsorbed multilayered film architecture formation (compare Figure 7 in [8]). The slope of *γ_LV_* cos *θ_A_* plotted as a function of *γ_LV_*, demonstrated in Figure 6c, made possible the surface surfactant excesses ratio: *Γ_SL_*/*Γ_LV_* to be evaluated for two cleansers (Corega Tabs, Kin Oro) and the mothrinses representative (Eurodont—right-hand scale). Generally, the shape of the dependences is similar despite the class of cleaning liquids considered. *Γ_SL_*/*Γ_LV_* ratios were derived for pre-micellar and post-micellar (corresponding to the practical treatment case) concentration ranges. As can be noticed, the dependence slopes (the *a* parameters) can be only determined outside the region of intermediate concentrations, where the structure of the adsorptive layer varies in an irregular way. The obtained *a* mean values were equal to −1.49 (Eurodont), −0.81 (Corega Tabs), −0.20 (Kin Oro) at (*C < CMC*) whereas attained −1.81 (Eurodont), −0.15 (Corega Tabs), −0.62 (Kin Oro) at (*C > CMC*). So-called conventional one-component surfactant water solutions exhibited negative slopes (from −0.33 to −0.17), which points to interfacial (PMMA–water) adsorption via nonpolar interactions [24].

### 4.5. Cleanser Surfactant Partitioning at LV and SL Interfaces

*Γ_SL_*/*Γ_LV_*, i.e., the surface surfactant excesses ratio was determined for all the cleansers over the whole concentration range. Values of *a* in contact with PMMA are summarized in Table 6. The data reported by others for differentiated in polarity model surfactant water solutions, together with the mouthrinse data, were already collected in Table 6 of our recent paper [7].

As mentioned, conventional surfactant adsorption at the PMMA/water interface is mediated by nonpolar interactions. They reveal absolute slopes value up to 0.3, which suggests that they are likely to tile at the interface with about 1/3 of the adsorption at the liquid/vapor interface. The slope of the linear dependence between the adhesional tension and surface tension appears to be negative (see Figure 2 in [25]). The slope is dependent on the kind of surfactants in the two-component mixture and its composition [25].

The surface excesses ratios obtained were in the range from −2.85 (Protefix) to −0.10 (Fitty Dent) at the pre-micellar concentration region and between −3.04 (Kurikur) and −0.15 (Corega Tabs) at *C > CMC*. Generally, the absolute slope ׀*a*׀ values were lower than −1, which evidences lower surface Gibbs’ adsorption of the adhered molecules at the *S*/*L* interface than at the *L*/*V* one but with a few exceptions, i.e., Kurikur, Protefix at *C < CMC* and Kurikur at *C > CMC*. Since the cleanser stands for a mixture of several compounds of differentiated surface activity, the net-tied, vertically segregated, multi-layered 3D molecular structures are likely to be created, as argued in [8]. Moreover, zwitterionic surfactants like betaines, which could be found in cleanser formulations [24], with their hydrophilic polar heads demonstrate unique adsorptive properties at a solid substratum. The betaine adsorption at the PMMA–liquid interface takes place through hydrophilic interaction where the alkyl chain is oriented upwards to the solution at a low bulk concentration, which leads to the lowering of adhesional tension. At higher concentrations (*C* >> *CMC*), the betaine molecules are adsorbed by a hydrophobic interaction mechanism at the solid substratum that makes the solid more hydrophilic (related to *γ_SV_*↑ and *θ*↓) and would improve adhesional tension [8]. Such an unconventional surface wettability evolution was noticed for a few of the studied cleanser products.

### 4.6. Microbial Adhesion vs. Substratum Wettability

There is a link between the solid surface wettability signatures and antimicrobial adhesion efficiency reported in the literature. In order to limit the adhesion of bacteria communities to the polymer surface, many signatures of substratum characteristics have to be registered, like the roughness architecture, surface charges, and surface free energy, as postulated in [26]. The roughness of materials is an important factor since irregularities increase and due to the amount of microorganisms remaining on the denture surface after the cleaning process. Water CAs of 40–70° were found to enhance the microbial cells’ colonization degree and adhesion, whereas surfaces with CAs of 54–130° exhibited the higher adsorption efficiency of bacterial peptidoglycan. The polar component of *γ_SV_* was found to be important for cell adhesion and spreading. In particular, values greater than 15 mJ m^−2^ promoted spreading, but a polar component lower than 5 mJ m^−2^ led to reduced cell spreading. A solid SFE decrease was revealed for acrylic denture resins after storage in substances for the denture’s hygiene [27]. Eventually, hydrophobic materials with *γ_SV_* of 20–30 mJ m^−2^ exhibit lower bacterial adhesion if referred to polar substrata.

Surfaces of moderate wettability have a higher capacity to bind bacterial cells in reference to highly hydrophobic (CA >> 90°) or hydrophilic (CA << 90°) surfaces. A further way to limit bioadhesion is to create a superhydrophobic surface (CA > 150°) by incorporating roughness to surfaces of low surface energy. For such a system, where a liquid is in contact with the solid surface, air becomes entrapped between roughening features. Thus, the incorporation of entrapped air plays a decisive role in suppressing bacteria adhesion by limiting the contact area decreasing adhesion forces. A Cassie–Baxter model of CA for such a surface was proposed [21]. Among the available denture cleansers exhibiting bactericidal and fungicidal properties, one of the most widely applied disinfectants appears to be sodium hypochlorite (0.5% and 1% NaClO) [28]. Its microbial destructive mechanism originates from the occurrence of chloride (Cl^−^) and hydroxide (OH^−^) ions that affect microbial cell wall damage and degrade fatty acids and lipids. Chlorhexidine gluconate is another compound commonly found in cleanser and mothrinse products, which binding to negatively charged sites of the bacterial cell wall leads to cell wall disruption [29].

### 4.7. Marangoni Spreading Kinetics and Penetration Coefficient

A surface tension-mediated flow directed to regions of high surface tension referred to as the Marangoni mechanism is a transport process occurring in a liquid layer deposited at the solid substratum. Its velocity $U_{S}$ (see Equation (7)) is attributed to the thermally induced surface tension gradients [13]. The penetration coefficient *PC* (see Equation (6)) is a quantity reflecting the ability of a liquid to penetrate into a capillary gap, like pores or gingival pockets. The *PC* is determined from the *γ_LV_*, contact angle *θ*, and viscosity *μ* data of the cleanser–MMA surface system [3]. Both values of *PC* and $U_{S}$ evaluated here are summarized in Table 7. *PC* values are contained in a rather narrow range from 7.03 (Corega Tabs) to 8.28 (Kin Oro)·10^−2^ m s^−1^, lower than for pure water, whereas $U_{S}$ ones appear to be slower by a factor of 20–28, comparable to the data revealed by the mouthrinse–PMMA systems [7].

### 4.8. Cleanser vs. Mouthrinse Surface Signatures Comparison

Significant differences can be noticed in the surface characteristics key parameters of both the cleanser and mouthrinse formulations in contact with the dental polymer products used in oral cavity hygiene. The functional correlation between the adhesion–adsorption–wettability parameters and the viability of the oral plaque microbes is needed to elucidate in order to test cleanser’s antimicrobial efficiency. The surface parameters variability for both mentioned groups of cleaning liquids are compared in Table 8. Since the chemical makeup of these two groups of substances differ significantly, only a general trend of surface adhesion–wettability signatures variability can be demonstrated.

The tests carried out on the adhesion and wettability of polymers in contact with cleansers do not allow to point to the most effective liquid in the process of destroying the bacterial flora on the material of dental prostheses in everyday clinical practice. However, individual hygiene fluids are characterized by the following characteristic values of the tested surface parameters, distinguishing them from others studied here and qualifying them for specific applications and locations of their operation in the interfacial system.

In particular, the following can be noticed:

1. The highest capacity in terms of the area size of the surface covered (lowest θ = 28.1, Kurikur);
2. The highest spreading and penetration speeds *PC* = 8.28·10^−2^ m s^−1^ (Kin Oro) and $U_{S}$ = 0.37·10^−2^ m s^−1^ (Protefix);
3. The lowest relative micellar concentration (*CMC*) = 0.017 (Kin Oro);
4. The lowest surface tension *γ_cmc_* (for *C = CMC*) = 44.3 mN m^−1^(Kurikur);
5. The highest saturation adsorption *Γ_max_* = 4.71·10^6^ mol m^−2^ (Protefix);
6. The highest surface activity *σ ~ Kp* = 537 mN m^−1^ (Kurikur);
7. The maximal degree of the structural complexity of the adsorption layer at the *L*/*V* interface ~*S_S_* = 0.076 mN m^−1^ K^−1^ (Fitty Dent);
8. The highest adhesional tension =30.4 mN m^−1^ (Kin Oro) as a measure of the surface energy (*γ_SL_ = γ_SV_ − γ_LV_* cos *θ_A_*) variability;
9. The most hydrophobic polymer in contact with *γ_SV_* = 26.2 mJ m^−2^ (Corega Tabs);
10. The maximal surface excess ratio (for *C > CMC*), i.e., the maximum molecular accumulation at the *L*/*S* interface *a* = −3.04 (Kurikur).

## 5. Conclusions

The partitioning coefficient of *K_P_* seems to be an important parameter, with low values pointing to the more soluble compound in the aqueous phase, thus being easily available for contact with microorganisms in an antibacterial treatment. The ratio *γ_LV_* cos *θ_A_*/*W_A_* is a significant parameter of wettability energetics being related to the adhesive strength removal ability of the cleanser liquids. CA studies revealed the following trend in the wettability parameters variability of the studied cleanser/dental polymer system referred to as the PMMA/pure water one: *θ*, *θ_A_* and *θ_R_*↓, *CAH*↑, *Π*↓, *W_A_*↓, *γ_SV_*↓, $\gamma_{SV}^{p}$, and (*γ_LV_* cos *θ_A_*)↑. The surfactant excesses ratios were negative, which exhibits a hydrophobic surface interaction mechanism at *S*/*L* interfaces, but their high absolute values revealed the creation of sandwich-like, multilayered vertically segregated structures in the cleanser mixtures consisting of differentiated surface activity compounds. The cleanser solution concentration *C*_0_, recommended by the producer in dental polymer treatment practice, appeared to be several times higher than these required to obtain the saturated molecular adsorptive layer at the interfaces of the air/solution/solid system, i.e., *C*_0_ >> *CMC*. The cleanser product’s successive dissolution with pure water was reflected in the following wettability parameters variability: *θ_A_* and *θ_R_*↑, *CAH*↓, *Π*↑, *W_A_*↑, *γ_SV_*~const.,$\;\gamma_{SV}^{p}$↑, and (*γ_LV_* cos *θ_A_*)↓, which could affect the antimicrobial efficiency of the product.

Further factors of concern in the elimination methodology of oral cavity dental plague; i.e., spreading speeds turned out to be *PC* = (7.03–8.22)·10^−2^ m s^−1^ and much lower, and $U_{S}$ = (0.13–0.37)·10^−2^ m s^−1^ induced from the Marangoni temperature surface tension gradient-driven flow. The measurement of surface adsorption and adhesion parameters on a solid polymer in contact with a multicomponent mixture of cleanser formulations allows for determining the optimal concentration and a place of accumulation of key components for antibacterial activity in the bulk liquid phase and elimination of oral cavity biofilm formation at the surface of the prosthetic material. In particular, *K_P_* and *Γ_LV_* are indicators of the efficiency of particular active substance accumulation in the planktonic phase, while *γ_SV_*, a = *Γ_SL_*/*Γ_LV_*, and *W_A_* reflect the degree of accumulation of the required active substance at the surface of the immersed polymer. To sum up, this work demonstrates a promising path to control wettability in a dental polymer/cleanser system useful in optimizing the cleaning agent makeup.

## Figures and Tables

**Table 1:** Physical and surface characteristics of commercial cleanser solutions. Experimental uncertainties and standard deviations from the mean are given in brackets.

Liquid	*C*_0_ [mg L^−1^] (0.1)	*γ_LV_* [mN m^−1^] (0.2)	*ρ*·10^3^ [kg m^−3^] (0.07)	*µ* [mPa s] (0.05)	pH [-] (0.1)	Product Source
Corega Tabs	10.7	36.2	0.92	1.03	7.9	Stafford Miller, Dungarvan, Ireland
Fitty Dent	54.1	33.4	0.97	1.13	9.0	FittyDent Int. Pinkafeld, Austria
Kin Oro	11.1	35.6	0.93	1.10	7.8	Bartex, Poznan, Poland
Kurikur	13.3	34.0	0.99	1.05	8.7	Burstenmann GMBH, Stutzengrun, Germany
Protefix	28.3	33.8	0.91	1.15	9.1	Queiser, Flensburg, Germany
Water	-	72.5	0.96	0.89	6.8	Milipore, Burlington, MA, USA

**Table 2:** Surface adsorptive parameters of cleansers at air/liquid interface. Mean values and standard deviations (given in brackets).

Liquid	*CMC* [*C*/*C*_0_]	*γ_CMC_* [mN m^−1^]	*Γ_max_*·10^6^ [mol m^−2^]	*A_lim_* [nm^2^ molec^−1^]	*|σ|* [mN m^−1^]
Corega Tabs	0.072 (0.01)	48.3 (0.2)	1.16 (0.04)	140.1 (4.5)	424 (15)
Fitty Dent	0.064 (0.02)	48.3 (0.2)	3.38 (0.06)	49.5 (2.6)	433 (16)
Kin Oro	0.017 (0.01)	55.9 (0.2)	2.54 (0.05)	65.3 (1.8)	561 (25)
Kurikur	0.088 (0.03)	44.3 (0.2)	2.85 (0.07)	61.6 (2.6)	537 (28)
Protefix	0.043 (0.01)	54.3 (0.2)	4.71 (0.09)	35.2 (1.9)	302 (16)

**Table 3:** Surface tension, entropy, and entropic term contributions to the total surface enthalpy at *C* = *C*_0_, *T* = 25 °C. Mean and standard deviation values (in brackets).

Liquid	*γ_LV_* [mN m^−1^] (0.2)	*S_S_* [mN m^−1^ K^−1^] (0.01)	*T S_S_* [mN m^−1^] (0.2)	*H_S_* [mN m^−1^] (0.4)
Corega Tabs	36.2	0.113	33.3	69.5
Fitty Dent	33.4	0.076	22.4	55.8
Kin Oro	35.6	0.082	24.2	59.8
Kurikur	34.0	0.061	18.0	52.0
Protefix	33.8	0.183	54.0	87.8
Water	72.5	0.163	48.1	120.6

**Table 4:** Wettability energetics parameters for cleanser liquid–PMMA system at *C = C*_0_. Mean and standard deviation values (in brackets). Values of *γ_LV_* from Table 1 were used. Young, static contact angle *θ* derived from the relation: cos *θ* = ½ cos *θ_A_* + ½ cos *θ_R_* [10].

**Liquid**	***Θ*** **[°]** **(1°)**	***Θ_A_*** **[°]** **(1°)**	***Θ_R_*** **[°]** **(1°)**	***CAH *** **[°]** **(2°)**	***П*** **[mN m^−1^]**
Corega Tabs	40.0	53.3	20.7	32.6	12.2 (0.5)
Fitty Dent	32.7	42.2	19.6	22.6	6.7 (0.3)
Kin Oro	23.9	31.5	12.5	19.1	4.4 (0.2)
Kurikur	28.1	33.9	21.0	12.9	3.5 (0.2)
Protefix	28.2	30.8	25.4	5.4	1.5 (0.1)
Water	64.6	76.0	52.0	24.0	26.9 (1.1)
**Liquid**	***W_A_*** **[mJ m^−2^]**	***γ_LV_* cos *θ_A_*** **[mN m^−1^]**	***γ_SV_*** **[mJ m^−2^]**	***γ_SV_^d^*** **[mJ m^−2^]**	***γ_SV_^p^*** **[mJ m^−2^]**
Corega Tabs	57.4 (1.1)	21.7 (0.2)	26.2 (1.4)	23.1 (1.2)	3.1 (0.2)
Fitty Dent	58.1 (1.1)	24.7 (0.2)	27.4 (1.4)	25.3 (1.2)	2.1 (0.2)
Kin Oro	66.0 (1.2)	30.4 (0.2)	31.9 (1.5)	30.6 (1.3)	1.3 (0.2)
Kurikur	62.3 (1.1)	28.3 (0.2)	30.3 (1.5)	28.5 (1.3)	1.8 (0.2)
Protefix	62.9 (1.1)	29.1 (0.2)	31.1 (1.5)	29.2 (1.3)	1.9 (0.2)
Water	89.5 (1.4)	10.2 (0.1)	38.9 (1.9)	27.8 (1.3)	11.1 (0.6)

**Table 5:** Wettability parameters for Corega Tabs–PMMA system versus cleanser relative concentration *C*/*C*_0_ = (1/2)*^n^*, where *n* = 0, 1, 2…11); *C*_0_—original product concentration, *CMC* = 0.072. The data set closest to the *CMC* concentration is marked with a bold font. Mean and standard deviation values (in brackets).

*C*/*C*_0_ = (1/2)*^n^*, *n*	*Θ_A_* [°] (1°)	*Θ_R_* [°] (1°)	*CAH* [°] (2°)	*П* [mN m^−1^]	*W_A_* [mJ m^−2^]	*γ_SV_* [mJ m^−2^]	*γ_SV_^d^* [mJ m^−2^]	*γ_SV_^p^* [mJ m^−2^]	*γ_LV_* cos *θ_A_* [mN m^−1^]
0	53.3	20.7	32.6	12.2 (0.5)	57.4 (1.1)	26.2 (1.4)	23.1 (1.2)	3.1 (0.2)	21.7 (0.2)
1	55.7	20.5	35.2	13.8 (0.5)	58.0 (1.1)	25.9 (1.4)	22.7 (1.2)	3.3 (0.2)	20.9 (0.2)
2	56.6	22.2	34.4	14.5 (0.5)	59.7 (1.1)	26.6 (1.4)	23.2 (1.2)	3.9 (0.2)	21.2 (0.2)
3	57.5	30.0	27.5	13.8 (0.5)	64.6 (1.1)	29.2 (1.5)	24.9 (1.3)	4.3 (0.2)	22.6 (0.2)
**4**	**62.4**	**27.9**	**34.5**	**18.4 (0.6)**	**64.1 (1.1)**	**28.0 (1.4)**	**23.4 (1.2)**	**6.6 (0.2)**	**20.3 (0.2)**
5	68.4	28.5	39.8	22.9 (0.7)	61.3 (1.1)	25.9 (1.4)	21.0 (1.1)	7.1 (0.3)	16.5 (0.2)
6	72.4	37.9	34.5	23.5 (0.7)	63.0 (1.1)	26.5 (1.4)	20.5 (1.1)	7.3 (0.3)	14.6 (0.1)
7	73.5	40.1	33.4	24.1 (0.7)	64.5 (1.1)	27.2 (1.4)	20.7 (1.1)	9.4 (0.3)	14.3 (0.1)
8	73.6	45.1	28.5	22.3 (0.7)	67.6 (1.2)	29.0 (1.5)	21.7 (1.1)	10.7 (0.4)	14.9 (0.1)
9	77.7	48.4	29.3	24.4 (0.7)	65.7 (1.2)	27.7 (1.4)	19.9 (1.1)	8.5 (0.3)	11.6 (0.1)
10	78.0	48.7	29.3	26.1 (0.8)	69.7 (1.2)	29.3 (1.5)	21.0 (1.1)	9.1 (0.4)	12.0 (0.1)
11	79.4	50.2	29.2	28.1 (0.8)	72.9 (1.3)	30.6 (1.5)	21.6 (1.1)	8.9 (0.3)	11.3 (0.1)

**Table 6:** *Γ_SL_*/*Γ_LV_* for the cleanser/water solution/PMMA interfacial system for pre-micellar and post-micellar concentration ranges. Mean values and standard deviations (in brackets).

Liquid	*Γ_SL_*/*Γ_LV_*	
	*C < CMC*	*C > CMC*
Corega Tabs	−0.81 (0.32)	−0.15 (0.06)
Fitty Dent	−0.10 (0.03)	−0.62 (0.17)
Kin Oro	−0.20 (0.08)	−0.62 (0.17)
Kurikur	−1.16 (0.44)	−3.04 (2.91)
Protefix	−2.85 (0.88)	−0.21 (0.08)

**Table 7:** Penetration coefficients *PC* and Marangoni spreading speeds $U_{S}$ for cleanser–PMMA systems at *C = C*_0_. Quantity error in brackets.

Liquid	*PC*·10^−2^ [m s^−1^]	$U_{S}$·10^−2^ [m s^−1^]
Corega Tabs	7.03 (0.15)	0.25 (0.04)
Fitty Dent	7.13 (0.17)	0.16 (0.02)
Kin Oro	8.28 (0.19)	0.17 (0.03)
Kurikur	7.63 (0.16)	0.13 (0.07)
Protefix	7.57 (0.18)	0.37 (0.09)
Water	9.80 (0.20)	0.80 (0.09)

**Table 8:** Comparison of surface characteristics parameters, general trends, for the studied groups of cleaning denture liquids (cleansers) and (mouthrinse); comprehensive data from [7]. Conditions: *C = C*_0_, *C > CMC* at *T* = 25 °C; units as throughout this study. Denotations: higher (>), lower (<), and comparable (~) on average.

Surface Parameter	Cleansers	>, <, ~	Moutrinses
**Surface adsorption**			
*γ_LV_(C_0_*	33.4–36.2	~	29.7–39.9
*CMC*	0.02–0.04	<	0.03–0.12
*Γ_max_*	1.16–4.71	>	0.24–0.97
*A_lim_*	35–140	<	170–839
*σ ~ K_P_*	302–561	<	230–1120
**Thermodynamics**			
*S_S_*	0.013–0.183	<	0.095–0.231
*H_S_*	52.0–87.8	<	66.8–97.6
**Contact angles**			
*θ*	28.1–40.0	<	30.2–46.1
*θ_A_*	30.8–53.3	<	42.2–65.2
*θ_R_*	12.5–25.4	>	10.0–22.3
*CAH*	5.4–32.6	<	23.9–38.9
**Wetting energetics**			
*Π*	1.5–12.2	<	4.9–19.8
*W_A_*	57.4–62.9	~	50.4–67.3
*γ_LV_* cos *θ_A_*	21.7–30.4	~	18.4–28.7
*γ_SV_*	26.2–31.9	~	22.4–31.5
$\gamma_{SV}^{d}$	23.1–30.6	~	18.9–29.3
$\gamma_{SV}^{p}$	1.3–3.1	~	1.3–3.6
Excess ratio (*C > CMC*)	−0.15–−3.04	~<	−1.81
**Spreading Kinetics**			
*PC*	7.03–7.63	<	6.51–9.82
$U_{S}$	0.13–0.37	<	0.2–0.46

## Data Availability

The data presented in this study are available on request from the corresponding author. The data are not publicly available due to privacy.
